# Bilateral breast myxedema caused by Graves’ disease and responsive to multipoint subcutaneous injection of long-acting glucocorticoid: Case report

**DOI:** 10.1097/MD.0000000000026469

**Published:** 2021-06-25

**Authors:** Tingting Du, Wangyan Jiang, Hongchang Luo, Fuqiong Chen, Gang Yuan, Muxun Zhang, Zhelong Liu

**Affiliations:** a Department of Endocrinology, Tongji Hospital, Tongji Medical College, Huazhong University of Science and Technology, Wuhan, China, Branch of National Clinical Research Center for Metabolic Disease, Hubei; b Department of Ultrasonography, Tongji Hospital, Tongji Medical College, Huazhong University of Science and Technology, Wuhan, China.

**Keywords:** breast, case report, glucocorticoid, Graves’ disease, myxedema

## Abstract

**Rationale:**

With the absence of ophthalmopathy, thyroid dermopathy especially lesions at atypical locations is a very rare presentation. We herein report an original case of bilateral breast myxedema caused by Grave's disease.

**Patient concerns:**

A 21-year-old unmarried woman presented with a 4-month history of Grave's disease and a 1-month history of progressive bilateral breast enlargement. She had symmetrical bilateral breast enlargement with redness and nonpitting thickening of the skin, diffusely enlarged thyroid glands, and no exophthalmos.

**Diagnosis:**

Ultrasonography, magnetic resonance imaging scan, and skin biopsy confirmed the diagnosis of bilateral breast myxedema.

**Interventions:**

The patient was treated with multipoint subcutaneous injections of triamcinolone acetonide in each breast every month.

**Outcomes:**

The bilateral breast returned approximately to its normal size after therapy for 6 months.

**Conclusions:**

Our case illustrates that multipoint subcutaneous injection of glucocorticoids is beneficial for bilateral breast myxedema.

## 1 Introduction

Thyroid dermopathy is a rare autoimmune manifestation of Grave's disease resulting from a local autoimmune response in cutaneous connective tissues. It is defined by the deposition of mucinous substances in the reticular dermis.^[1]^ Thyroid dermopathy together with hyperthyroidism and exophthalmos constitute the classical triad of Graves’ disease.^[2]^ Due to its dependent position and increased exposure to mechanical stress, the pretibial area is the most typical site involved.^[1]^ Thus, thyroid dermopathy is commonly called pretibial myxedema. However, thyroid dermopathy can also occur in other sites such as areas exposed to repeated trauma, surgical scars, vaccination sites, burn scars, skin graft, and affect other areas, including face, pinna, shoulder, abdomen, upper back, head, neck, upper extremities and buttock.^[1–6]^ We herein report a case of bilateral breast myxedema caused by Grave's disease who was successfully treated with multipoint subcutaneous injection of a long-acting glucocorticoid in 6 months of treatment. According to our literature review, no case of bilateral breast myxedema induced by Grave's disease has been reported. Hence, this case is original.

## 2 Case report

A 21-year-old unmarried woman presented to the outpatient department reported a 1-month history of progressive symmetrical bilateral breast enlargement accompanied by the presence of mild thickening of the skin in the bilateral breast. There was no pain but occasional pruritus in the bilateral breast area. An 8 o’clock biopsy specimen of the left breast showed no evidence of malignant involvement. This patient was referred to our department for further management. On eliciting a detailed history, she had a 4-month history of Grave's disease and had been taking propylthiouracil 100 μg 3 times a day for approximately 3 months. Suppressed thyrotropin-stimulating hormone (TSH) levels, as well as elevated free triiodothyronine (FT3) and thyroxine (FT4) levels, were noted at the time of the diagnosis of Grave's disease.

**Figure 1:**
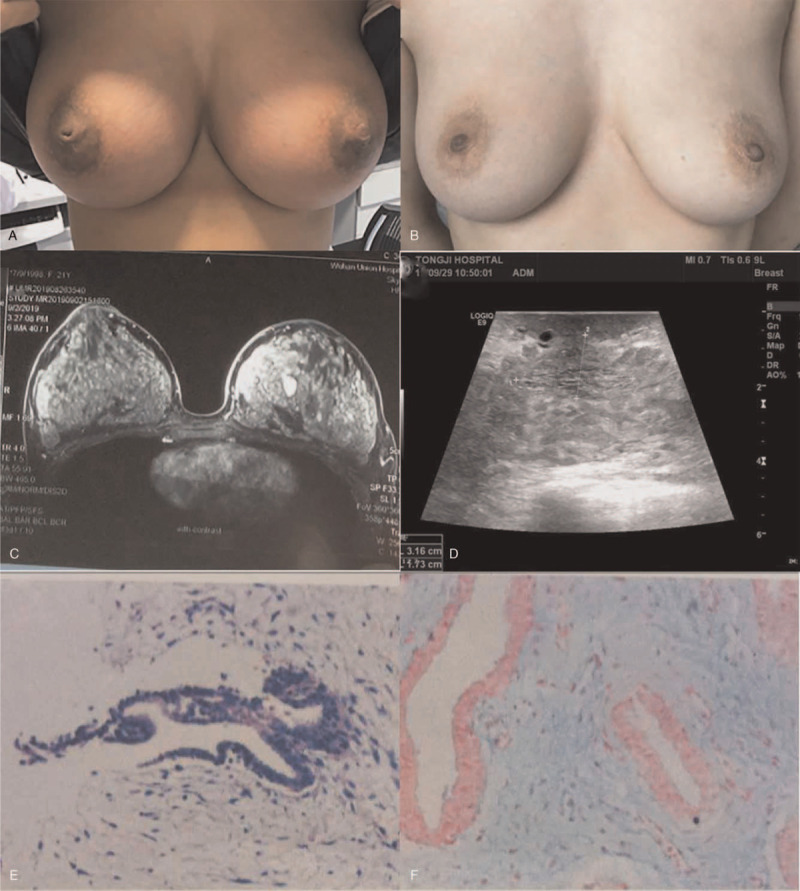
(A): The patient's symmetrical bilateral enlargement before therapy, redness, and nonpitting thickening of the skin involved nearly the entire bilateral breast. (B): The bilateral breast returned approximately to its normal size after therapy for 6 months, the color of the skin return to normal. C-1D: A magnetic resonance imaging scan (C) and ultrasonography (D) showing marked dermal edema before therapy. (E–F): Histopathological examination of bilateral breast tissues on alcian blue staining revealed abundant deposition of glycosaminoglycans in these lesions before therapy.

On physical examination, she had diffusely enlarged thyroid glands, grade 2 without bruits. Breast examination confirmed the symmetrical bilateral enlargement. Redness and nonpitting thickening of the skin involved nearly the entire bilateral breast. There were focal areas of hyperpigmentation and hyperkeratosis involving the bilateral areola (Fig. 1A). Galactorrhoea was not found. A 2.0∗2.5 cm nodule located in the 8 o’clock of the left breast and a 1.0∗1.5 cm nodule located in the 10 o’clock of the right breast were palpable. Ophthalmic examination revealed no exophthalmos or retraction of the upper eyelids.

Laboratory investigations revealed as follows (reference ranges shown parenthetically): normal FT3 4 pg/mL (2.0–4.4), normal FT4 11.97 ng/L (9.32–17.09), but suppressed TSH, 0.121 uIU/ml (0.27–4.2), which indicated that this patient attained a subclinical hyperthyroidism state after taking 3-month propylthiouracil. Thyroid peroxidase antibodies and thyroglobulin antibodies were normal: 20.14 IU/mL (0–34) and 36.14 IU/ml (0–115), respectively. However, Thyroid-stimulating antibodies were strongly positive: 36.74 IU/L (0– 1.58). Serum follicle-stimulating hormone 1.98 mIU/mL (1.79–5.12), luteinising hormone 2.73 mIU/mL (1.2–12.86), estradiol 169 pg/mL (49–291), prolactin 20.87 ng/mL (3.34–26.72), and testosterone concentration < 0.01 ng/mL (≤0.75) were all in normal range and consistent with her menstrual cycle. Sex hormone-binding globulin was 82.5 nmol/L (18.2–135.5). Anti-nuclear antibodies, anti-microsome antibodies, tumor marker, complete blood count, kidney and liver panels were normal. Taken together, there was no evidence of malignancy or systemic disease except for Graves’ disease. A magnetic resonance imaging scan of the bilateral breast (Fig. 1C) and ultrasonography (Fig. 1D) showed marked dermal edema and confirmed the increased thickness of bilateral breast myxedema. Biopsy of the bilateral breast (8 o’clock in the left breast and 10 o’clock in the right breast) was performed and histopathological examination of these tissues on Alcian blue staining revealed abundant deposition of glycosaminoglycans in these lesions (Fig. 1E-F), and a diagnosis of thyroid dermopathy was established.

**Figure 2:**
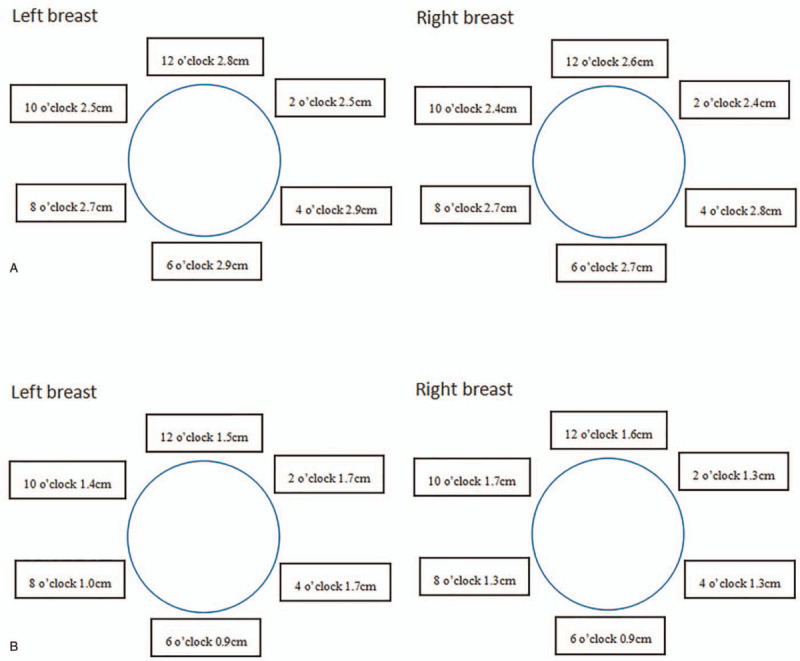
(A): Ultrasonography showing marked fluid sonolucent area before therapy. (B): The fluid sonolucent area of the bilateral breast decreased significantly after therapy for 6 months. All the measuring sites were 3 cm apart from the nipple.

Then she received multipoint subcutaneous injections of a solution of 5 mg/mL of triamcinolone acetonide monthly. For each treatment session, a total volume of 8 mL was administered in 30 to 40 injection sites in each breast. The injections were started from the areola and then moved to the borderline of the breast. Each injection point was 2 cm apart. The diameter of the needle was 0.5 mm. The depth of needle insertion was 1.0 cm. There was a thinning, softening, and normal color of skin in the bilateral breast 1 month after the first injection. The response was observed uniformly to all treated areas. And the size of the bilateral breast became profoundly smaller. This was proved by the ultrasonography examination in which significantly reduced fluid sonolucent area across the entire breast was noted (Fig. 2). As the treatment courses were continued, the size of the bilateral breast gradually became smaller. The bilateral breast returned approximately to its normal size after 6 months of treatment with a total dosage of 480 mg triamcinolone acetonide for the bilateral breast (Fig. 1B). No adverse effects from the use of the corticosteroid were observed. During this triamcinolone treatment, she was treated with propylthiouracil for thyrotoxicosis using a dose-titration regime. The patient was followed up every 1 or 2 months in the outpatient department of our hospital. The bilateral breast remained its normal size without thickening of the skin. The last thyroid function test in May 2021 showed that TSH, FT3, and FT4 levels were in the normal range.

## 3 Discussion

The association between thyroid function level and breast disorders has been well documented.^[7]^ However, bilateral breast myxedema has never been reported in association with thyroid disease. Epidemiological studies have reported an increased risk of breast cancer in women with hyperthyroidism.^[8,9]^ Gynecomastia and breast necrosis have been noted in patients with hyperthyroidism.^[10,11]^ In our case, biopsy specimens of the bilateral breast showed no sign of malignant involvement or necrosis. Gynecomastia or mastoplasia is usually developed in men and can resolve with successful management of hyperthyroidism.^[10,11]^ Our case developed her bilateral breast enlargement after she got improvement in her thyroid function state. In addition, no skin involvement is seen in patients with gynecomastia. Reddish and nonpitting thickening of the skin in the bilateral breast was observed in our case. Hence, our case of bilateral breast enlargement was not gynecomastia. Our report gives new evidence of the association between thyroid disorders and breast disorders.

Thyroid dermopathy is an extrathyroidal manifestation of Grave's disease. Almost all cases of thyroid dermopathy have coexisting ophthalmopathy.^[2]^ Generally, thyrotoxicosis appears first, followed by ophthalmopathy and then dermopathy much later.^[2]^ However, this case represents a rare initial presentation of thyroid dermopathy in the absence of preceding clinical ophthalmopathy. Although thyroid dermopathy classically manifested on the pretibial area, involvement may be localized elsewhere on the body. Our case illustrates an involvement of the area in the bilateral breast, which represents an extremely rare finding. Myxedema must be considered when evaluating patients with known Grave's disease.

The pathogenesis of thyroid dermopathy involves activation and proliferation of fibroblast triggered by autoimmunity to the TSH receptor, which provokes a cascade of immune processes and overproduction of glycosaminoglycans.^[1,2]^ There is evidence that trauma and injury may initiate or exaggerate the presence of thyroid dermopathy.^[1,2,6]^ Our case illustrated that her breast enlargement was more evident on the left than on the right after undergoing a second biopsy on the left side.

Thyroid dermopathy is diagnosed based on serological thyroid hormone abnormalities, typical skin involvement, and high levels of TSH receptor antibodies.^[1,2]^ In the presence of ophthalmopathy and pretibial lesions, the diagnosis of thyroid dermopathy is clear.^[1,2]^ However, with the absence of ophthalmopathy, isolated thyroid dermopathy especially lesions at atypical locations is a very rare presentation and represents a diagnostic challenge. Skin biopsy is needed in this situation.^[1,12]^

Although some lesions of thyroid dermopathy are asymptomatic or simply cause cosmetic problems, therapy should be started early to prevent secondary processes such as lymphatic microcirculation obstruction and fibrosis.^[13]^ The treatments include management of risk factors (weight management and tobacco cessation), rapid normalization of thyroid function, and management of dermopathy and associated ophthalmopathy.^[1]^ Local corticosteroid therapy, compressive therapy and complete decompressive physiotherapy, systemic immunomodulation, octreotide therapy, and surgical excision can be employed to manage dermopathy.^[1]^ Due to a lack of uniform and normative standard treatment, the management of thyroid dermopathy remains a therapeutic challenge. Multipoint subcutaneous injection of glucocorticoids is so far the most common and effective treatment for pretibial myxedema.^[6,14,15]^ In the index case, complete remission of the bilateral breast myxedema together with minimum adverse effects were noted after 6 sessions of multipoint subcutaneous injection of glucocorticoids. This case indicates that multipoint subcutaneous injection of glucocorticoids is a safe and effective treatment for this problem.

## Acknowledgments

We thank the patient and her family for participating in this study.

## Author contributions

**Data curation:** Fuqiong Chen.

**Formal analysis:** Hongchang Luo.

**Supervision:** Zhelong Liu.

**Writing – original draft:** Tingting Du, Wangyan Jiang.

**Writing – review & editing:** Gang Yuan, Muxun Zhang.
